# Impact of a new diagnostic pathway for prostate cancer—from systematic transrectal to targeted MRI fusion transperineal biopsies

**DOI:** 10.1007/s11255-025-04875-7

**Published:** 2025-11-03

**Authors:** Sara Vejlgaard Kristensen, Martin Wirenfeldt, Katrine Thelle, Else Brohm Kallestrup, Mads Hvid Poulsen

**Affiliations:** 1 Department of Urology, Esbjerg Hospital, University Hospital of Southern Denmark, Finsensgade 35, 6700 Esbjerg, Denmark; 2 Department of Pathology, Esbjerg Hospital, University Hospital of Southern Denmark, Esbjerg, Denmark; 3 Department of Radiology and Nuclear medicine, University Hospital of Southern Denmark, Esbjerg, Denmark; 4 Department of Regional Health Research, University of Southern Denmark, Odense, Denmark; 5 BRIDGE (Brain Research—Inter Disciplinary Guided Excellence), Department of Clinical Research, University of Southern Denmark, Odense, Denmark

**Keywords:** Prostate cancer, MRI, Transperineal biopsy, Transrectal biopsy

## Abstract

**Purpose:**

Advancements in prostate cancer diagnostics have changed the diagnostic pathway from traditional ultrasound-guided systematic transrectal biopsies to pre-biopsy prostate multiparametric MRI and targeted transperineal biopsies. This study aims to determine changes in prostate cancer detection rates, clinical outcomes, and treatment before (2018) and after (2023) implementing the new diagnostic pathway.

**Methods:**

This retrospective study comprises two cohorts from two Danish hospitals—one from 2018 (*n* = 266) and one from 2023 (*n* = 304). In each cohort, men with suspected prostate cancer who underwent biopsies were analysed. Data were obtained from medical records. Categorical variables were compared using Chi-square or Fisher’s exact test. Continuous variables were analysed with the Mann–Whitney *U* test in STATA version 18. *P* value ≤ 0.05 was considered significant.

**Results:**

The 2023 cohort showed a significantly higher detection of clinically significant prostate cancer (Gleason ≥ 3 + 4) compared to the 2018 cohort (65.41% vs. 41%, *p* < 0.01). Median Gleason scores increased from 6 to 7 (IQR 6–7 vs. 6–8, *p* < 0.01). Hospital admissions decreased from 11.3% to 3% (*p* < 0.01) in the 2023 cohort. Treatment and D’Amico’s risk groups showed no significant changes. Limitations include potential selection bias from only including biopsied patients and findings limited to two Danish hospitals, reducing generalizability.

**Conclusion:**

Transitioning to targeted transperineal biopsies improved cancer detection and reduced complications. While the Gleason score increased, risk groups and treatment plans remained unchanged. These results support implementation in clinical practice, though further studies are needed to assess long-term outcomes and broader applicability.

## Introduction

Prostate cancer is the second most commonly diagnosed cancer among men worldwide and the most frequently diagnosed cancer among men in Denmark [1]. Recent advancements in prostate cancer (PCa) diagnostics have introduced a new diagnostic pathway to address overdiagnosis and overtreatment of low-risk cases. This new approach also minimises complications associated with traditional transrectal biopsies [2–4].

Traditionally, the standard diagnostic procedure was systematic ultrasound-guided transrectal prostate biopsies (TR-Bx). This procedure involves a 12-core systematic sampling of prostate tissue through the rectum, guided by transrectal ultrasound imaging. The samples were taken from various regions of the prostate, particularly the peripheral zones [3]. However, this method underestimates PCa incidence, with a false negative rate of up to 49% [5]. Additionally, TR-Bx frequently causes minor complications, such as pain and bleeding. Severe infectious complications, however, remain the leading cause of hospitalisation after the procedure, despite antimicrobial prophylaxis [4, 6, 7].

The new diagnostic pathway integrates multiparametric magnetic resonance imaging (mpMRI) of the prostate and targeted ultrasound-guided transperineal biopsies as a replacement for TR-Bx. Previous studies have shown that transperineal biopsies (TP-Bx) outperform traditional TR-Bx. TP-Bx makes sampling of the anterior prostate easier and has a lower risk of complications, such as bleeding and infections [7, 8]. mpMRI is used before biopsies to identify suspicious areas. These MRI lesions are graded using Prostate Imaging-Reporting and Data System (PI-RADS) and then used to guide biopsies [9–11]. The change of the pathway with the introduction of mpMRI of the prostate prior to biopsy, and the use of targeted only biopsied, were based upon a number of pivotal papers showing better clinical outcome [12–14]. The combination of mpMRI with targeted biopsies is associated with a 57% improvement in the detection of clinically significant PCa (Gleason ≥ 3 + 4) and a reduction in clinically insignificant PCa of 57% [3, 12]. This combination can reduce the number of biopsy cores needed and avoid unnecessary biopsies [2, 15, 16].

Several systematic reviews and randomised clinical trials have compared traditional systematic biopsies to mpMRI-guided biopsies [10, 13, 16, 17]. Research comparing transrectal and transperineal biopsies has also been done [7, 18, 19]. However, minimal research has focused on the impact of the complete change from systematic TR-Bx to mpMRI-targeted TP-Bx in a day-to-day clinical setting outside clinical trials.

This retrospective cohort study on men with suspected prostate cancer aims to determine the impact of the transition from traditional systematic transrectal biopsies to the new diagnostic pathway, incorporating prostate MRI and targeted transperineal biopsies. The study compares two cohorts of men from two Danish regional hospitals servicing the same population before (2018) and after (2023) implementing the new diagnostic pathway. Specifically, the study is comparing:

1. Prostate cancer detection rates and pathological cancer staging (Gleason score, ISUP-grade).
2. Clinical outcomes (hospital admission, death).
3. Changes in the initial treatment plans following diagnosis.

## Material (patients) and methods

### Study population

Two cohorts comprise the basis of this retrospective observational study, one from 2018 and one from 2023. In each cohort, men with suspected prostate cancer who underwent prostate biopsies were included. The patients in the two cohorts were from five specific municipalities serviced by two Danish regional hospitals. Patient lists for the two cohorts were generated using biopsy procedure codes. Men in the two cohorts with a prior PCa diagnosis were excluded.

**Fig. 1:**
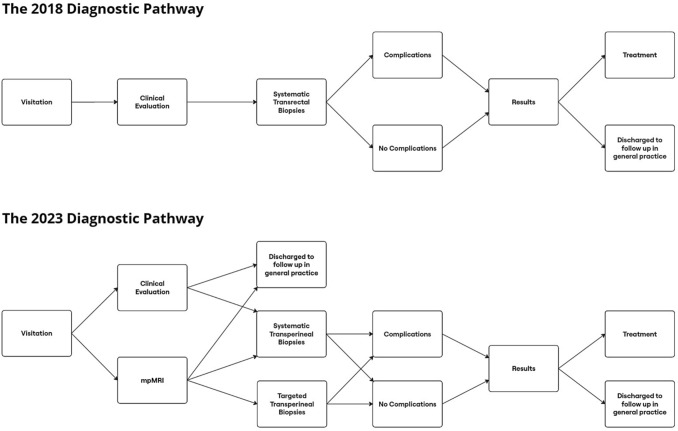
Flowchart illustrating the two diagnostic pathways

The 2018 cohort included 266 men who underwent biopsies as part of the old diagnostic pathway. They were from a population of 239,060 people. The 2023 cohort comprised 304 men out of a population of 239,124 people. They followed the new pathway. The two pathways are illustrated in Fig. 1.

The study received approval from the Regional Scientific Ethical Committee.

### Imaging

The mpMRI examinations were all performed using a 3.0 T scanner (Magnetom Vida, Siemens Healthineers) with a phased-array coil around the pelvic area. If a 3.0 T MRI was contraindicated due to implants, either a 1.5 T scan (Magnetom Aera, Siemens Healthineers) or no MRI was conducted. The mpMRI protocol included tri-planar T2-weighted, diffusion-weighted, and dynamic contrast-enhanced imaging as recommended by ESUR (European Society of Urogenital Radiology) [20]. All imaging was reviewed by subspecialized radiologists according to the PI-RADS V2.1 guidelines [9]. Target lesions were contoured using MIM software (MIM Software Inc.). For men with multiple lesions, the highest PI-RADS score was used for further analysis.

### Biopsies

All biopsies were done as outpatient procedures according to local guidelines.

The mpMRI-TP-Bx was performed under local anaesthesia (10 ml lidocaine 1%) with an 18-gauge biopsy needle. Three to five biopsy cores were taken targeted from each suspicious lesion. The targeting was done using a biplane ultrasound probe rectally and fusing the mpMRI prostate contours with ultrasonic imaging using software with an integrated fusion system. The cores from each target lesion were preserved and documented separately [21]. For those with contraindications to MRI or high clinical suspicion (≥ cT3 cancer), 12 systematic transperineal biopsies were performed instead.

The non-targeted systematic TR-Bx were performed under local anaesthesia with an 18-gauge biopsy needle. Ten to twelve cores were taken rectally, guided by ultrasound using a triplane ultrasonic probe. The TR-Bx were performed under antibiotic prophylaxis (pivmecillinam and amoxicillin with clavulanic acid one hour before the procedure and continued for three days after).

Experienced pathologists at the Pathology Department at Esbjerg Hospital reviewed all biopsy cores. Each biopsy core length was noted. If PCa was detected, the cancer fraction and Gleason score for each core were also reported. Gleason-score was based on the highest histological grade detected. Clinically significant prostate cancer (csPCa) was defined as Gleason ≥ 3 + 4 = 7 or International Society of Urological Pathology (ISUP) grade ≥ 2 [13, 22].

### Study data

Demographic info and clinical characteristics were recorded from visitation records and journal entries from the initial hospital consultation. The mpMRI descriptions were collected from radiology reports and biopsy diagnoses from pathology reports. Clinical outcomes of hospital admissions, death and treatment were collected from patient records. Hospital admissions were recorded up to 14 days following the biopsies, and death was recorded up to 30 days after. For data analysis, treatment plans were summarized into the following groups: Active Surveillance, Watchful Waiting, Curative intended and Life-prolonging. Risk groups were generated using the EAU prostate cancer guidelines originally based on D’Amico’s risk classification [23, 24]. All data were collected retrospectively during September and October 2024 and stored in a secure database.

### Statistical analysis

The data were analysed using STATA version 18.0 [25]. Patient characteristics were stratified by year. Categorical variables were presented as absolute and relative frequencies. Continuous variables were recorded as means and standard deviations (SDs), with mean difference and 95% confidence intervals (95% CI). Pearson’s Chi-square and Fisher’s exact test were used to compare the proportions of categorical variables. Continuous variables were tested for normal distribution using the Mann–Whitney *U* test. A *p* value of ≤ 0.05 was considered significant.

## Results

### Patient characteristics

There were no significant differences between the two cohorts regarding patient characteristics, except for mean age at referral, biopsy type and MRI status. The latter two are due to the changes in the diagnostic pathway. The mean age increased significantly from 69.9 years in the 2018 cohort to 71.9 years in the 2023 cohort (*p* = 0.01). The patient characteristics are summarised in Table 1.

**Table 1:** Patient demographics and clinical characteristics

Year				
	2018	2023	Mean difference (95% CI)	*P* Value
N	256 (46.72%)	292 (53.28%)		
Age	69.9 (8.64)	71.89 (8.6)	−1.97 (−3.42 to −0.52)	0.01*
PSA	77.07 (494.99)	114.52 (698.1)	−37.44 (−140.32 to 65.43)	0.47
Prostate volume	57.89 (30.56)	60.72 (33.12)	−2.83 (−8.22 to 2.56)	0.30
PSA-density	0.82 (2.33)	2.22 (13.44)	−1.40 (−3.08 to 0.28)	0.10
DRE				
Normal	139 (54.30%)	152 (52.05%)	−0.02 (−0.11 to 0.01)	0.60
Suspicious	117 (45.70%)	140 (47.95%)		
Biopsy type				
Targeted TP-Bx	0 (0%)	189 (64.73%)		
Targeted TR-Bx	4 (1.56%)	0 (0%)		
Systematic TP-Bx	0 (0%)	98 (33.56%)		
Systematic TR-Bx	252 (98.44%)	5 (1.71%)		
MRI				
No	251 (98.05%)	101 (34.59%)		
Yes	5 (1.95%)	191 (65.41%)		

1 All results were reported as N (%) or Mean (SD). *95% CI* 95% Confidence Interval. * Significant < 0.05. *DRE* Digital Rectal Exam

### Cancer detection rate and staging

The cancer detection rate was significantly higher in 2023 than in 2018 (79.1% vs. 54.7%, *p* < 0.01; Table 2). The median Gleason score rose from 6 in 2018 to 7 in 2023 (*p* < 0.01). The distribution of ISUP grades is summarised in Table 2. The detection of csPCa was significantly higher in the 2023 cohort (65.4%) compared to the 2018 cohort (41%, *p* < 0.01). There was no significant difference in risk groups between the cohorts (p = 0.46).

**Table 2:** Cancer

Year				
	2018	2023	OR (95% CI)	*P* Value
Cancer				
No	116 (45.31%)	61 (20.89%)	3.37 (2.22–5.12)	< 0.01*
Yes	140 (54.69%)	231 (79.11%)		
Clinically significant cancer				
No	151 (58.98%)	101 (34.59%)	3.15 (2.07–4.71)	< 0.01*
Yes	105 (41.02%)	191 (65.41%)		
Gleason-score	4.02 (3.77)	5.85 (3.15)	−1.83 (−2.41 to −1.25)	< 0.01*
ISUP-grade				
Benign	116 (45.31%)	61 (20.89%)	3.37 (2.22–5.12)	< 0.01*
1	35 (13.67%)	10 (13.70%)		
2	36 (14.06%)	75 (25.68%)		
3	16 (6.25%)	40 (13.70%)		
4	21 (8.20%)	27 (9.25%)		
5	32 (12.50%)	49 (16.78%)		
Risk group				
Low-risk	21 (12.07%)	22 (8.59%)	1.11 (0.58–2.14)	0.46
Intermediate-risk	64 (36.78%)	103 (40.23%)		
High-risk	89 (51.15%)	131 (51.17%)		

2 Gleason-score is reported as Mean (SD) with Mean difference (95% CI). *OR* Odds Ratio. *95% CI* 95% Confidence Interval. The OR is adjusted for age, PSA and prostate volume. * Significant < 0.05

### Clinical outcomes and treatment

In the 2018 cohort, 29 men (11.3%) had post-biopsy complications necessitating emergency department visits due to infection or bleeding. This number decreased significantly to nine patients (3%) in the 2023 cohort (adjusted OR = 0.23, 95% CI = 0.12–0.5, *p* < 0.01). There were no fatalities following the biopsies among the included patients in 2018. One man (0.34%, *p* = 0.35) in 2023 died following hospitalization with sepsis shortly after biopsy; although temporally related, a direct causal link to the biopsy could not be firmly established.

The initial treatment plan was noted for all included patients diagnosed with PCa. The different treatment tactics included curative intended, active surveillance, watchful waiting and life-prolonging treatment. There was no significant change in the initial treatment plan between the cohorts (*p* = 0.89).

## Discussion

In this retrospective cohort study, both the detection rate of csPCa and the fraction of csPCa/PCa were significantly higher in the 2023 cohort using the new diagnostic pathway compared to the 2018 cohort using the old diagnostic pathway (Table 2). There was a significant difference in pathological staging but not in risk groups, PSA levels, or digital rectal exams. The study found a significant reduction in post-biopsy admissions in the 2023 cohort compared to the 2018 cohort, but no significant changes in mortality following the biopsies. Looking at the initial treatment plans, no significant changes were found between the cohorts. These results indicate that the new diagnostic pathway involving a prebiopsy mpMRI and targeted TP-Bx improves the detection of clinically significant prostate cancer while also improving the patients’ clinical outcomes following the biopsies.

The increased detection rate of PCa, especially csPCa, in the 2023 cohort aligns with multiple previous studies supporting the use of mpMRI to improve diagnostic accuracy by targeting clinically significant PI-RADS lesions [16, 17, 26]. However, it is important to consider the potential influence of the Will Rogers phenomenon [27] on these findings: It has previously been shown that MRI-targeted biopsies may lead to an upshift in Gleason score without an actual change in the prognosis [28, 29]. Thus, diagnostic upstaging does not necessarily imply improved survival outcomes. In our study, despite higher Gleason scores in the 2023 cohort, risk group distribution and initial treatment plans remained largely unchanged. We did not directly control for the Will Rogers phenomenon beyond comparing baseline clinical characteristics between cohorts; therefore, some of the observed differences in pathological staging may reflect stage migration rather than a true increase in disease severity.

The increase in detection rate of csPCa may also be partially attributed to the change in the region sampled from TR-Bx to TP-Bx. Transperineal biopsies have an increased ability to sample the anterior and apical regions of the prostate and, in general, sample the prostate from another angle, which may result in increased detection rates. However, studies comparing the two approaches have shown mixed results. Some studies find a higher detection rate of csPCa using the transperineal approach, while others do not find a significant difference in detection rates between the two [7, 18, 19].

Overall, the detection rate of PCa went up from 140 men in the 2018 cohort to 231 men in the 2023 cohort, an increase of 65%; meanwhile, the population over the 5 years was stable. The change in PCa detection should be seen in light of the increased public awareness regarding prostate cancer and increased referral rates from primary care due to enlarged opportunistic screening [30–32]. This could explain the higher number of biopsied patients in the 2023 cohort. A larger referred population screened with mpMRI would likely also result in increased PCa detection rates. Previous studies estimate that using mpMRI allows 27% of referred men to avoid biopsies [10, 17]. This aligns with the difference in PCa detection rates in Table 2. Applied to our cohorts, the estimated referred population in the 2018 cohort would be 258 men and 362 men in the 2023 cohort, an increase of 40% in referrals. Thus, the new referral patterns account for 2/3 of the rise in the cancer detection rate, while the remaining 1/3 may be driven by the new diagnostic pathway. We acknowledge that these changes in referral practices represent a potential confounder, which limits direct comparisons between the two cohorts.

The significant reduction in admissions from the 2018 cohort to the 2023 cohort was attributed to the change from TR-Bx to TP-Bx in the new diagnostic pathway. Previous literature has found that TP-Bx is associated with a lower risk of complications such as infection and bleeding compared to TR-Bx [7, 8].

The reduction in hospital admissions, fewer benign biopsies and increased detection rates of PCa and especially csPCa, may lead to potential savings in health care costs. Fewer hospital admissions due to complications should translate directly into lower costs associated with post-biopsy care. Additionally, the incorporation of prebiopsy mpMRI may help screen patients to avoid unnecessary biopsies, while also improving detection rates of csPCa, which lowers diagnostic costs over time. Very few cost-effectiveness studies have been done, so more studies on the long-term economic benefits of this new diagnostic pathway are warranted [33].

## Strengths and limitations

Strengths include the clear delineation between the two full cohorts based on diagnostic pathways, ensuring minimal crossover. The consistency of hospital systems, protocols, departments, and personnel across the two cohorts enhances the internal validity of the findings and minimizes variability from external influences. Furthermore, the exclusive use of patient journals and reports as data sources avoids recall bias and ensures that the information collected accurately reflects what happened. This also allows for recording outcomes such as mortality and deceased patients, which would have been impossible relying on patient-reported data.

Certain limitations must also be acknowledged. First, only people biopsied were included due to differences in referral codes between 2018 and 2023; this introduces a selection bias, particularly in the 2023 cohort, where mpMRI enabled many men to avoid biopsy altogether. As a result, our study likely underestimates the true impact of mpMRI on reducing unnecessary biopsies. Including all men referred on suspicion of PCa would have provided a more comprehensive view of the performances of the two diagnostic pathways.

In addition, because the study population came from two Danish regional hospitals, the generalizability of our findings to other healthcare systems may be limited.

Despite these limitations, the study provides insight into the impact of incorporating mpMRI and targeted TP-Bx into the diagnostic pathway for prostate cancer. The new pathway have been implemented nationwide in Denmark up till now. The future brings further optimization of the pathway: Biparametric MRI is under evaluation as is micro-ultrasound (microUS) as an emerging alternative to MRI [34–37]. Future research should aim to validate these findings in other healthcare settings and include longer follow-up periods to better understand the long-term implications of including prebiopsy MRI and targeted transperineal biopsies.

## Conclusion

This study determines that transitioning from traditional systematic ultrasound-guided transrectal biopsies to the new diagnostic pathway—using mpMRI for selecting and targeting transperineal biopsies—improved prostate cancer detection and reduced post-biopsy hospitalizations. However, a substantial rise in referrals in 2023 likely contributed to the increased detection. These findings emphasize the effectiveness of improving clinical outcomes in diagnosing prostate cancer with the new diagnostic approach.

## Data Availability

The data cannot be shared openly to protect study participant privacy.
